# Automatic recognition of topic-classified relations between prostate cancer and genes using MEDLINE abstracts

**DOI:** 10.1186/1471-2105-7-S3-S4

**Published:** 2006-11-24

**Authors:** Hong-Woo Chun, Yoshimasa Tsuruoka, Jin-Dong Kim, Rie Shiba, Naoki Nagata, Teruyoshi Hishiki, Jun'ichi Tsujii

**Affiliations:** 1 Department of Computer Science, Graduate School of Information Science and Technology, The University of Tokyo, Tokyo, Japan; 2 School of Computer Science, University of Manchester, UK; 3 Japan Biological Information Research Center, Japan Biological Informatics Consortium, Japan; 4 Biological Information Research Center, National Institute of Advanced Industrial Science and Technology, Japan; 5 SORST, Japan Science and Technology Corporation, Japan; 6 National Centre for Text Minig (NaCTeM), Manchester, UK

## Abstract

**Background:**

Automatic recognition of relations between a specific disease term and its relevant genes or protein terms is an important practice of bioinformatics. Considering the utility of the results of this approach, we identified prostate cancer and gene terms with the ID tags of public biomedical databases. Moreover, considering that genetics experts will use our results, we classified them based on six topics that can be used to analyze the type of prostate cancers, genes, and their relations.

**Methods:**

We developed a maximum entropy-based named entity recognizer and a relation recognizer and applied them to a corpus-based approach. We collected prostate cancer-related abstracts from MEDLINE, and constructed an annotated corpus of gene and prostate cancer relations based on six topics by biologists. We used it to train the maximum entropy-based named entity recognizer and relation recognizer.

**Results:**

Topic-classified relation recognition achieved 92.1% precision for the relation (an increase of 11.0% from that obtained in a baseline experiment). For all topics, the precision was between 67.6 and 88.1%.

**Conclusion:**

A series of experimental results revealed two important findings: a carefully designed relation recognition system using named entity recognition can improve the performance of relation recognition, and topic-classified relation recognition can be effectively addressed through a corpus-based approach using manual annotation and machine learning techniques.

## Introduction

This paper presents an information recognition system for gene-disease association mentioned in literature. Such systems are receiving increased attention, particularly from medical doctors and pharmacists, as they have the potential of reducing the burden on researchers to explore the extensive pool of literature.

Similar works include those by Rosario and Hearst [1] in which they classified seven semantic relations between entities *disease *and *treatment *using several machine learning techniques, including *hidden Markov models *and *neural networks*. The relations were *cure*, *only disease*, *only treatment*, *prevent*, *vague*, *side effect*, *and no cure*. Some of these semantic relations described binary relations between diseases and treatments. Using 3,662 labeled sentences in MEDLINE abstracts and dynamic hidden Markov models, the authors achieved an F-measure of 0.71.

In our previous work [2], we extracted disease-gene relations using dictionaries and a named entity filtering technique. We used the following features: the target entity, unigram and bigram words of the target entity, and the presence of capital letters, numbers, Greek letters, and affixes in the target entity. There is a disadvantage in the size of the corpus: only 1,000 co-occurrences (sentences) were used for training and testing procedures. They achieved 78.5% precision and 87.1% recall.

We aim to recognize relations between prostate cancer terms and relevant gene terms from MEDLINE abstracts. To determine the utility of this approach, we identified prostate cancer and gene terms with ID tags that are used in six publicly available biological databases. Moreover, to enable human genetics experts and oncologists to use our results, we classified them and their relations based on six topics. We call this approach *topic-classified relation recognition*.

## Topic-classified relation recognition

Our system first collects sentences that contain at least one pair of gene and prostate cancer terms, using dictionary-based longest matching. Dictionary-matching results contained numerous false positive gene and prostate cancer terms and their relations, so we used machine learning (ML)-based named entity recognition (NER) and topic classified relation recognition to solve this problem. Our system outputs topic-classified relations.

### Construction of human gene and disease dictionaries

To link each output gene or prostate cancer term to publicly available biomedical databases, we created human gene and disease dictionaries by merging the entries of numerous public biomedical databases. These dictionaries provide gene- and disease-related terms and cross-references between the original databases.

#### The human gene dictionary

A unique *LocusLink *identifier for genetic loci is assigned to each entry in the human gene dictionary, which enabled us to consistently merge gene information contained in different databases. Each entry in the merged gene dictionary holds all relevant literature information associated with a given gene. We used five public databases to build the gene dictionary: *HUGO*, *LocusLink*, *SwissProt*, *RefSeq*, and *DDBJ *(July 2004). Each entry in the merged gene dictionary consists of five attributes: gene name, gene symbol, gene product, chromosomal band, and PubMed ID tags. The current version of the gene dictionary contains a total of 34,959 entries with 19,815 HUGO-approved gene symbols, 19,788 HUGO-approved gene names, and 29,470 gene products. Note that there are numerous alias gene symbols and gene names in these entries.

#### The disease dictionary

We used the Unified Medical Language System (UMLS) to collect disease-related vocabulary. From the 2003AC edition of the UMLS Metathesaurus, we selected 12 unique identifiers of semantic types (TUIs) that correspond to disease names, abnormal phenomena, or symptoms (Table 1). From these TUIs, we extracted 431,429 unique identifiers for strings (SUIs) and stored them as a disease-related lexicon. Therefore, this disease dictionary is not specific to prostate cancer.

### Annotation of corpus

To build training and testing sets, we collected 1,362,285 abstracts through a MEDLINE search using 248 prostate cancer-related terms selected from our disease dictionary. From these abstracts, we generated 2,503,037 co-occurrences using dictionary-based longest matching. When a sentence contained more than one gene term and more than one prostate cancer term, the system made sufficient copies of the sentence to accommodate all possible gene-prostate cancer term pairs. We call these copies *co-occurrences*, which are the input units of our system. We chose 3,939 co-occurrences randomly, and they were annotated by four biologists.

The types of annotation in our corpus are the following:

• Gene and prostate cancer named entities:

To begin with, these terms were recognized by dictionary-based longest matching, and biologists annotated whether given gene and prostate cancer terms were correct.

• Relations between entities:

Biologists annotated whether a binary relation existed between entities.

• Classification of gene and prostate cancer terms and their relations based on topics:

We classified gene and prostate cancer terms and their relations based on 13 topics: *study description (method), modality, genetic variation, epigenetics, gene expression, gene produces variation, molecular function, sub-cellular localization, pharmacology, clinical marker, risk factor, tumor biology, and remarks*.

• PMID:

Our corpus provides a PMID for each co-occurrence.

For the annotation of binary relations between gene and prostate cancer terms, the biologists considered three aspects.

1. Pathophysiology, mechanisms of prostate cancer, including etiology, causes of prostate cancer.

2. Therapeutic significance of genes or gene products; specifically, classification of genes or gene products based on their current therapeutic use and their potential as therapeutic targets.

3. Use of genes and gene products as markers for prostate cancer risk, diagnosis, and prognosis.

#### Six topics

In addition to the *binary relation *between gene and prostate cancer terms, we classified prostate cancer and gene terms and their relations based on 13 topics. All topics are mutually independent, so certain co-occurrence can be classified by more than one topic. We selected the following six topics based on the inter-annotator agreement rates that had over 70% F-measure. To calculate the inter-annotator agreement rates for the four annotators, we randomly selected 40 co-occurrences and annotated them.

Examples of topics contain gene and prostate cancer terms that are represented by *G *and *P*, respectively, with square brackets.

1. Study description (method)

Sentences in the *Methods *section of papers do not give specific results or conclusions. However, those sentences might still contain allusive gene-prostate cancer term relations.

**Example 1 ***Thereafter plasma S, cortisol (F) and *[adrenocorticotropic hormone]_*G *_*(ACTH) responses to metyrapone were investigated in 13 normal adult males and 39 patients with *[prostatic cancer]_*P*_.

2. Genetic variation

There are genotypic differences among individuals in a population. For example, mutation (including germ line and somatic), polymorphism (SNP, microsatellite, restriction fragment length), and LOH.

**Example 2 ***A polymorphism in *[endostatin]_*G*_, *an angiogenesis inhibitor, predisposes for the development of *[prostatic adenocarcinoma]_*P*_.

3. Gene expression

Gene expression is the phenotypic manifestation of a gene by the processes of genetic transcription and translation. Its profiling is also included.

**Example 3 ***The expression of *[HNK-l]_*G *_*antigen on *[prostatic cancer]_*P *_*was investigated immunohisto-chemically using the avidin-biotin-peroxidase complex (ABC) method with the anti-HNK-1 monoclonal antibody*.

4. Epigenetics

Chemical mutations to DNA or histones alter the structure of a chromatin without changing the nucleotide sequence of the DNA.

**Example 4 ***Hypermethylation of the 5' promoter region of the *[glutathione S-transferase pi]_*G *_*gene (GSTP1) occurs at a very high frequency in *[prostate adenocarcinoma]_*P*_.

5. Pharmacology

Pharmacology is the science of drugs, including their compositions, uses, and effects.

**Example 5 ***OBJECTIVES: To assess the involvement of calcitonin gene-related peptide *([CGRP]_*G*_) *in the occurrence of hot flashes in men after castration for treatment of *[prostate cancer]_*P*_, *we investigated the effects of CGRP on skin temperature in surgically and medically castrated male rats*.

6. Clinical marker

Measurable and quantifiable gene products are used as biological parameters to assess health- and physiology-related factors, such as prostate cancer risk, prostate cancer diagnosis, cell line development, and epidemiologic studies.

**Example 6 ***The use of *[prostate specific antigen (PSA)]_*G *_*and digital rectal examination (DRE) results in a three fold increase in *[prostatic carcinoma]_*P *_*detection*.

### ML-based NER

We used ML-based NER for two purposes: to provide a feature for each candidate relation in an ML-based topic-classified relation recognition method and to filter out numerous false positive gene and prostate cancer terms from the dictionary matching results before performing topic-classified relation recognition. Maximum entropy (ME) models [3] have been developed and used to train the named entity (NE) filter. They exhibited good performances in the JNLPBA-2004 of biomedical NER [4] and the CoNLL-2003 shared task of NER [5], and they have been widely used in solving classification problems.

#### Features of NER

The following features were used in the NER.

• Bag of words:

All contextual terms in a co-occurrence.

• Candidate entities:

Candidate gene and prostate cancer terms that were recognized using dictionary matching.

• Unigram and bigram words of candidate entities:

Unigram words refer to the word before and after the candidate term; bigram words refer to the two adjacent words before and after the candidate term.

• Use of capital letters in the candidate term:

We determined whether the given entities consisted entirely of upper or lowercase letters or a combination of them.

• Use of numbers in the candidate term:

We determined whether the given entities contained numbers.

• Affixes of the candidate term:

We considered whether the given entities include the 11 biomedical suffixes: ~ *cin*, ~ *mide*, ~ *zole*, ~ *lipid*, ~ *rogen*, ~ *vitamin*, ~ *blast*, ~ *cyte*, ~ *peptide*, ~ *ma*, *and *~ *virus*.

• Greek letters in the candidate term:

We determined whether the given entities contained Greek letters (e.g., *alpha*, *beta*, *α*, and *β*).

Table 2 lists the performance of NER. The first rows for gene and prostate cancer terms express the performance using dictionary matching (baseline). Note that our dictionaries do not include all gene and prostate cancer terms, thus, we could not calculate the *absolute recall *in this experiment. Instead, we used *relative recall *as a performance measure, which is calculated assuming the baseline method performs at 100% of this metric. In this approach, we are interested in how precise our system is at correctly identifying the relations, rather than how often it misses other meaningful ones. Thus, we focused on improving its precision.

For gene name recognition, the most important feature was candidate names. Using it, we achieved 95.0% precision in our NER for gene names (an increase of 10.6% over using dictionary matching). The next two most important features were the bag of words and the unigram words. We achieved 93.5 and 93.1% precisions, respectively, using these features. For the task of prostate cancer term recognition, dictionary matching generated very high performance. Thus, it slightly improved the precision.

### ML-based topic-classified relation recognition

Gene and prostate cancer term pairs co-occurring in a sentence have some potential relations. However, these co-occurring pairs also have numerous false positive relations. We developed an ME-based relation recognizer to filter out false positives.

#### Features for topic-classified relation recognition

The following features were used in the topic-classified relation recognizer.

• Bag of words:

All contextual terms in a co-occurrence.

• Candidate gene and prostate cancer entities:

Entities that were recognized using dictionary matching.

• Unigram and bigram words of candidate gene and prostate cancer entities:

We determined unigram words of candidate gene and prostate cancer entities simultaneously. For bigram words, we followed the same procedure as that for unigram words.

• Order of candidate entities:

We accounted for the order of candidate gene and prostate cancer terms in each co-occurrence. In other words, we determined whether a candidate gene term appeared before a candidate prostate cancer term in each co-occurrence.

Table 3 lists the performance of relation recognition. For recognition of relation, study description, and genetic variation, the most important feature was *bag of words*: omitting it, we achieved only 89.6, 59.7, and 73.7% precisions and 97.5, 29.5, and 46.4% relative recalls, respectively, in topic-classified relation recognition (decreases in precision of 1.1, 7.2, and 5.6%, respectively, compared with those achieved using all features).

For recognition of gene expression, epigenetics, pharmacology, and clinical marker, the *order of candidate entities *seemed to be the most important feature. Leaving out the *order of candidate entities *lead to most significant decrease: decreases of 1.4, 2.1, 3.1, and 0.7% from 73.4, 85.4, 65.7, and 77.4% precisions, respectively.

## Experimental results

Table 4 shows the results of all experiments. Numbers in the first column represent the number of cooccurrences classified based on corresponding topics. All topics and relation were mutually independent, so a co-occurrence can be classified by more than one topic and relation. We performed 10-fold cross validation to evaluate the systems and measured the precision and relative recall of the system for 3,939 co-occurrences.

We conducted eight experiments for topic-classified relation recognition. The inputs of the experiments were co-occurrences that contained at least one pair of gene and prostate cancer terms recognized by dictionary-based longest matching. The first experiment used only gene and disease dictionary-based longest matching. The second and third experiments used dictionary matching and ME-based NE filtering. The next five experiments used ME-based topic-classified relation recognition. The fourth experiment used only ME-based topic-classified relation recognition and did not use NER results. The fifth and sixth experiments used ME-based NER results as features for topic-classified relation recognition. The seventh and eighth experiments used ME-based NER results as a filtering measure. We compared the ME-based NER results with human-generated NER annotation results. Thus, the second, fifth, and seventh experiments used ME-based NER results on both training and testing procedures, which we call *automatic NER*, and the third, sixth, and eighth experiments used human-generated NER annotation results on both training and testing procedures (a gold standard), which we call *manual NER*. A series of experimental results showed that automatic NER is comparable to manual NER.

### Performance using dictionary matching (baseline)

The baseline experiment is very simple. We assumed that all gene-prostate cancer pairs recognized by dictionary-based longest matching had a relation.

### Performance using dictionary matching and an NE filter

We applied NER to filter out false positive gene and prostate cancer terms generated by dictionary matching, and we assumed that all the remaining gene-prostate cancer pairs had a relation. NE filtering improved the precision of all topic-classified relation recognitions at the cost of a small reduction in recall. We used the best combination of features based on the F-measure that had been obtained empirically for NER.

• Recognition of gene names:

Candidate names, unigram words, and presence of capital letters in the candidate term.

• Recognition of prostate cancer names:

Candidate names, unigram words, and presence of capital letters or Greek letters in the candidate term.

The performance of recognizing general *relations *in the cells in the first and second rows, and fourth column of Table 4 was unusually high. Manual analysis revealed that most correctly identified gene-prostate cancer pairs were identified as correct relations: 96.7% of 2,494 correctly identified gene-prostate cancer pairs had been identified as a correct relation.

### Performance using ML-based topic-classified relation recognition

We used ML for topic-classified relation recognition with the best combination of features based on the F-measure.

• Study description, genetic variation, gene expression, and pharmacology:

Bag of words, candidate gene and prostate cancer terms, unigram and bigram words, and order of candidate terms.

• Relation:

Bag of words, candidate gene and prostate cancer terms, unigram words, and order of candidate terms.

• Epigenetics:

Bag of words, unigram and bigram words and order of candidate terms.

• Clinical marker:

Candidate gene and prostate cancer terms, unigram and bigram words, and order of candidate terms.

Although the experiment did not consider NER results, the precision of ML-based topic-classified relation recognition was much better than that in the baseline experiment.

### Performance using ML-based topic-classified relation recognition and NER results as features

We used NER results as features in addition to the contextual features that we used in the fourth experiment. Experimental results showed that using NER results as features for topic-classified relation recognition improved the precision in the relation and four topics. We can thus infer that NER information is a cogent feature. For recognition of epigenetics, the performance in the automatic NER experiment was higher than that in the manual NER experiment. These results are statistically not significant because the number of correct epigenetics relations is only 53.

### Performance using ML-based topic-classified relation recognition and NER as a filter

NER results were used to filter out gene-prostate cancer pairs over-generated by dictionary matching. Topic-classified relation recognition modules were given only co-occurrences that remained after filtering. We used the same combination of features as those in the fourth experiment. Filtering with NER results improved the performance of topic-classified relation recognition more than using them as features for ML-based topic-classified relation recognition. Recognition of epigenetics led to the most significant increase in precision (2.4%) and recognition of genetic variation led to the next most significant increase in precision (0.8%).

## Conclusion

We have developed ML-based topic-classified relation recognizers between prostate cancer and gene terms. Six topics were used to classify prostate cancer and gene terms, and their relations. Simple dictionary-based longest matching was tested, which produced numerous false positive results. Annotated abstracts were then input to an ME-based ML module to train NER and relation recognizers. A comprehensive series of experiments revealed that the ML-based approach that used rich contextual features have the potential to improve the performance of topic-classified relation recognition. The effect of combining the recognizers was also investigated. The results were encouraging, and we are planning several extensions that include incorporating disambiguation [6] and deep syntactic parsing techniques [7,8]. Both classes of techniques have previously been applied successfully to several tasks, and we expect that incorporating such techniques will supplement our methods by providing appropriate treatment to polysemous terms and richer features of deep syntactic structure.

## Figures and Tables

**Table 1:** Selected TUIs.

T019	Congenital abnormality	T048	Mental or behavioral dysfunction
T020	Acquired abnormality	T049	Cell or molecular dysfunction
T033	Finding	T050	Experimental model of disease
T037	Injury or poisoning	T184	Sign or symptom
T046	Pathologic function	T190	Anatomical abnormality
T047	Disease or syndrome	T191	Neoplastic process

**Table 2:** Performance of NER.

Target Entities	Features							Precision (%)	Relative recall (%)
			
	1	2	3	4	5	6	7		
GENE								84.4	100.0
								93.5	95.4
								95.0	97.6
								93.1	93.3
								84.4	100.0
								84.4	100.0
								84.4	100.0
								84.4	100.0
								**95.8**	97.0

PROSTATE CANCER								99.2	100.0
								99.3	99.8
								99.3	100.0
								99.3	100.0
								99.2	100.0
								99.2	100.0
								99.2	100.0
								99.2	100.0
								99.3	100.0

*Note*: 1) Bag of words (all words in co-occurrence), 2) candidate gene and prostate cancer names, 3) unigram words, 4) presence of capital letters in candidate term, 5) presence of numerical digits in candidate term, 6) presence of Greek letters in candidate term, 7) presence of affixes of candidate term.

**Table 3:** Performance of relation recognition.

	Features					Precision (%)	Relative recall (%)
			
	Order of entities	Bag of words	Candidate entities	Unigram	Bigram		
Any relation						0.907	0.958
						0.908	0.957
						0.896	0.975
						0.902	0.957
						0.903	0.966
						0.909	0.961

Study description						0.669	0.567
						0.650	0.568
						0.597	0.295
						0.662	0.551
						0.659	0.552
						0.651	0.550

Genetic variation						0.793	0.691
						0.781	0.665
						0.737	0.464
						0.791	0.669
						0.796	0.687
						0.794	0.680

Gene expression						0.734	0.614
						0.720	0.620
						0.762	0.466
						0.733	0.612
						0.743	0.613
						0.735	0.612

Epigenetics						0.854	0.660
						0.833	0.660
						0.905	0.358
						0.857	0.679
						0.854	0.660
						0.854	0.660

Pharmacology						0.657	0.431
						0.626	0.442
						0.642	0.264
						0.643	0.419
						0.637	0.419
						0.647	0.433

Clinical marker						0.774	0.723
						0.767	0.728
						0.772	0.730
						0.768	0.678
						0.771	0.727
						0.772	0.719

**Table 4:** Experimental results.

			Baseline with NE filter			RR with NER (feature)		RR with NER (filter)	
					
Topic-classified Relations	(%)	Baseline w/o NER	Automatic	Manual	RR w/o NER	Automatic	Manual	Automatic	Manual
Any relation	P	81.1	91.8	96.7	90.9	91.5	97.0	**92.1**	97.1
(3196)	R	100.0	97.0	100.0	96.1	96.1	99.6	96.5	99.6
Study description	P	26.7	30.2	31.8	66.9	67.5	70.8	**67.6**	70.6
(1050)	R	100.0	97.2	100.0	56.7	57.6	63.0	62.9	62.9
Genetic variation	P	7.1	8.1	8.4	79.3	78.6	81.9	**79.4**	83.1
(278)	R	100.0	98.9	100.0	69.1	67.3	70.1	73.6	73.6
Gene expression	P	27.1	30.8	32.3	73.4	73.0	76.2	**73.5**	76.8
(1067)	R	100.0	97.4	100.0	61.4	61.4	64.5	63.5	64.9
Epigenetics	P	1.3	1.6	1.6	85.7	86.0	85.7	**88.1**	88.1
(53)	R	100.0	100.0	100.0	67.9	69.8	67.9	69.8	69.8
Pharmacology	P	9.1	10.3	10.9	65.7	**66.1**	66.7	63.7	67.2
(360)	R	100.0	96.1	100.0	43.1	44.7	45.0	44.4	45.3
Clinical marker	P	31.5	35.9	37.5	77.4	**77.9**	78.2	76.6	78.3
(1240)	R	100.0	97.8	100.0	72.3	73.2	74.0	73.6	75.4

*Notes*) Numbers in the first column: frequency of correct predictions, NER: ML-based NER, RR: ML-based topic-classified relation recognition, Automatic: experiments using ML-based NER results, Manual: experiments using human-generated NER annotation results, P: precision, R: relative recall.
